# Development of a Patient Decision Aid to Support Shared Decision Making for Patients with Recurrent High-Grade Glioma

**DOI:** 10.3390/ijerph19127396

**Published:** 2022-06-16

**Authors:** Helle Sorensen von Essen, Frantz Rom Poulsen, Rikke Hedegaard Dahlrot, Karin Piil, Karina Dahl Steffensen

**Affiliations:** 1 Department of Neurosurgery, Odense University Hospital, Kloevervaenget 47, Indgang 44, 46, 1. sal, DK-5000 Odense, Denmark; frantz.r.poulsen@rsyd.dk; 2 Clinical Institute, University of Southern Denmark, J.B. Winsloews Vej 19, DK-5000 Odense, Denmark; rikke.dahlrot@rsyd.dk; 3 BRIDGE (Brain Research-Interdisciplinary Guided Excellence), University of Southern Denmark, J.B. Winsloews Vej 19, DK-5000 Odense, Denmark; 4 Department of Oncology, Odense University Hospital, Kloevervaenget 19, DK-5000 Odense, Denmark; 5 The Danish Center for Particle Therapy, Palle Juul-Jensens Blvd. 99, DK-8200 Aarhus, Denmark; 6 Department of Oncology, Center for Cancer and Organ Diseases, Copenhagen University Hospital, Blegdamsvej 9, DK-2100 Copenhagen, Denmark; karin.piil@regionh.dk; 7 Department of Public Health, Aarhus University, Bartholins Allé 2, DK-8000 Aarhus, Denmark; 8 Department of Regional Health Research, Faculty of Health Sciences, University of Southern Denmark, J.B. Winsloews Vej 19, DK-5000 Odense, Denmark; karina.dahl.steffensen@rsyd.dk; 9 Center for Shared Decision Making, Region of Southern Denmark, Beriderbakken 4, DK-7100 Vejle, Denmark

**Keywords:** shared decision making, patient decision aids, decision support, patient involvement, family involvement, high-grade glioma, neuro-oncology

## Abstract

When high-grade gliomas recur, patients, their families, and clinicians face difficult medical decisions. There is no curable treatment, and the treatment options all come with a risk of complications and adverse effects. The patients are often cognitively affected, and they need tailored decision support. The objective of this study was to develop a patient decision aid (PtDA) targeted at patients with recurrent high-grade gliomas. Based on existing knowledge and the International Patient Decision Aids Standards, the PtDA was developed through an iterative process. The PtDA was alpha-tested by potential users to assess its acceptability and usability. The development team comprised three clinicians, two patients, two family members, and a researcher. The fifth version of the PtDA was submitted to the alpha test. Eleven patients, nine family members, and eleven clinicians assessed the PtDA and found it acceptable. Three changes were made during the alpha test. Most participants perceived the PtDA to prepare patients for decision making and improve consultations. The involvement of potential users was emphasized during the development and alpha test process. The PtDA was assessed as useful and acceptable by patients, family members, and clinicians in the decision-making situation of recurrent high-grade glioma.

## 1. Introduction

High-grade gliomas (HGG), classified as WHO grade III and IV tumors, are the most aggressive brain tumors [1]. The primary treatment is surgical resection, followed by concomitant radiotherapy and chemotherapy [2]. The treatment is not curative, and all patients are at a high risk of tumor recurrence [3]. 

Clinical decision making concerning which treatments the individual patient can be offered at disease recurrence depends on the specific characteristics of the tumor, the tumor location, previous treatment responses and side effects, age, and the patient’s performance status [3]. Research indicates that repeated surgery can prolong survival if all visible tumors can be resected [4,5,6]. The survival benefit is less evident if only a part of the tumor can be resected [4,5,6]. At the time of recurrent HGG, different types of systemic chemotherapy can also prolong patient survival and improve quality of life [3]. The choice of chemotherapeutic treatment depends on the patient’s previous treatment course and treatment response [3]. The treatment for recurrent HGG is less evidence-based than primary treatment [3]. Additionally, patients suffer from an extended range of symptoms caused by the tumor’s brain affection and the side effects of previous surgery, chemotherapy, and radiotherapy [7,8].

The uncertainty concerning treatment outcomes and the trade-offs between benefits and disadvantages make it essential to involve patients and include their values and preferences in the decision-making process [9]. Based on this, an increasing focus on patient involvement and shared decision making (SDM) is emerging within neuro-oncology, suggesting that neuro-oncology patients want to be involved in SDM regarding their treatment and care [10,11,12,13,14]. 

SDM is an approach that combines professional expertise and scientific evidence with the subjective values and preferences of the individual patient in a shared decision [15,16]. Patient decision aids (PtDAs) are helpful tools for supporting SDM with patients [17]. A PtDA can provide balanced and unbiased information about treatment options and their associated benefits and disadvantages, and it can support patients in reflecting on the values and preferences related to these options [17]. Using a PtDA can increase patient involvement in decision making and support a decision that aligns with the patient’s values and preferences [17,18]. A PtDA can be designed as an app, paper leaflet, video, or interactive digital solution [17]. 

For patients with recurrent HGG, involvement in SDM can be challenging due to reduced cognitive abilities, such as memory, concentration, understanding, and information processing [19,20,21]. Nevertheless, these patients are faced with complicated and preference-sensitive decisions [9] and they require tailored decision support [9,19,20,21,22]. The reduced decision-making capacity also translates into a dependency on family support and family involvement in decision making [7,8]. The research on SDM for patients with HGG is limited, however, and despite a thorough search, no decision support tools or PtDAs have been identified for this population [11,12,17,23].

To increase decisional support for HGG patients and acknowledge the critical role of family support, the objective of this study was to develop a PtDA that was considered useful and acceptable by patients, their families, and the involved clinicians.

## 2. Materials and Methods

The study design was guided by the “Systematic development process for patient decision aids” [24] in accordance with the International Patient Decision Aids Standards (IPDAS) [24,25]. The IPDAS framework aims to enhance the quality and effectiveness of PtDAs by establishing a shared evidence-informed framework with a set of criteria for improving their content, development, implementation, and evaluation. 

A multidisciplinary development team was established, and they selected the content for the PtDA through an iterative process. After developing the PtDA, we alpha-tested it with potential users to assess its acceptability and usability. 

### 2.1. Development

#### 2.1.1. The Development Team

We established a multidisciplinary developmental team to provide a balanced representation of relevant clinicians, patients, and family representatives. The patient and family representatives were recruited through the Danish Brain Tumor Association. The team comprised two HGG patients, two family members of HGG patients, one neurosurgeon (FRP), one oncologist (RD), one neurologist, and one nurse researcher (HSE). The clinicians involved were well recognized within their areas of expertise. Due to the COVID-19 pandemic, it was unfeasible for the development team to hold physical meetings with all participants. Instead, the collaboration took place through online discussions and one-on-one meetings. 

#### 2.1.2. The Generic Template

The PtDA presented in this article is based on a generic PtDA template, the “Decision Helper”, which was developed by the Centre for Shared Decision Making at Lillebaelt Hospital, Denmark [26]. The template was designed according to the IPDAS criteria. It is a paper-based PtDA containing a preparation sheet that can be sent to patients in advance to prepare them for decision making and an in-consult PtDA consisting of a folder that presents the five steps of the decision-making process. Inside the folder are the potential treatment and care options described on individual option cards [26]. Each option card presents the benefits and disadvantages of the specific option, described in short sentences and supplemented by pictograms [26]. The template also contains a card with patient narratives related to the decision of interest to help patients reflect on their values and preferences while reading about the considerations and decisions of previous patients. 

**Figure 1:**
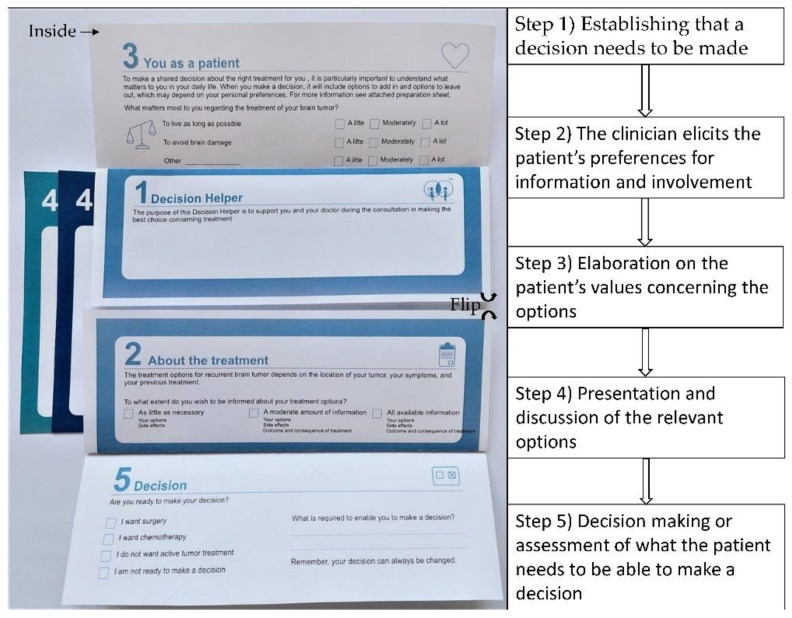
The in-consult patient decision aid and a description of the five steps in the decision-making process.

The five steps described in the template function as a guide for the clinician and help structure the consultation to increase patient and family involvement, support the clinicians’ verbal information, and strengthen SDM (Figure 1) [26].

#### 2.1.3. Selecting Content for the PtDA

**Figure 2:**
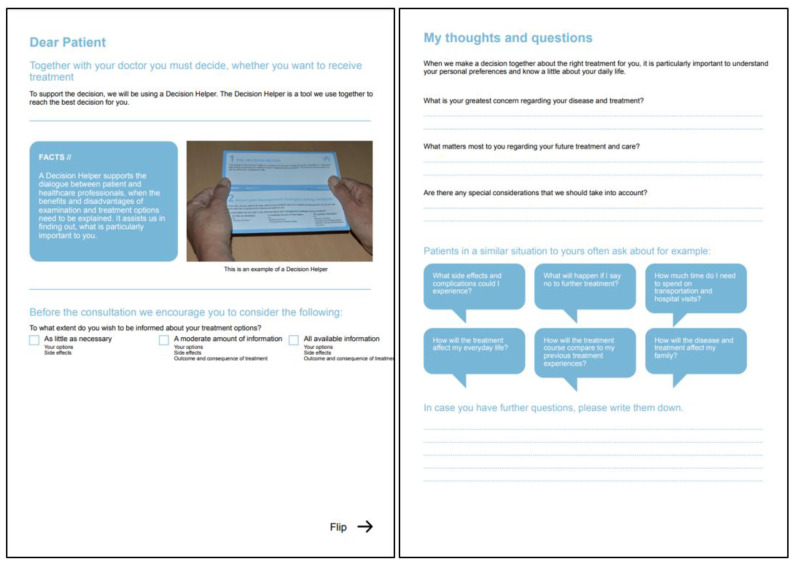
The preparation sheet to be handed out to patients before a decision-making consultation.

Step 1. To support future patients when they are considering treatment and care, we identified a list of questions typically asked by patients and family members. We selected the questions by recording, transcribing, and analyzing 10 decision-making consultations in the neurosurgical outpatient clinic. By applying a directed content analysis [27] carried out in NVivo (QSR International Pty Ltd., version 11.4, Warrington, UK), we identified all questions asked by patients and family members and grouped the questions into themes. The development team discussed the questions, and one representative question was formulated for each theme and presented on the preparation sheet (Figure 2).

**Figure 3:**
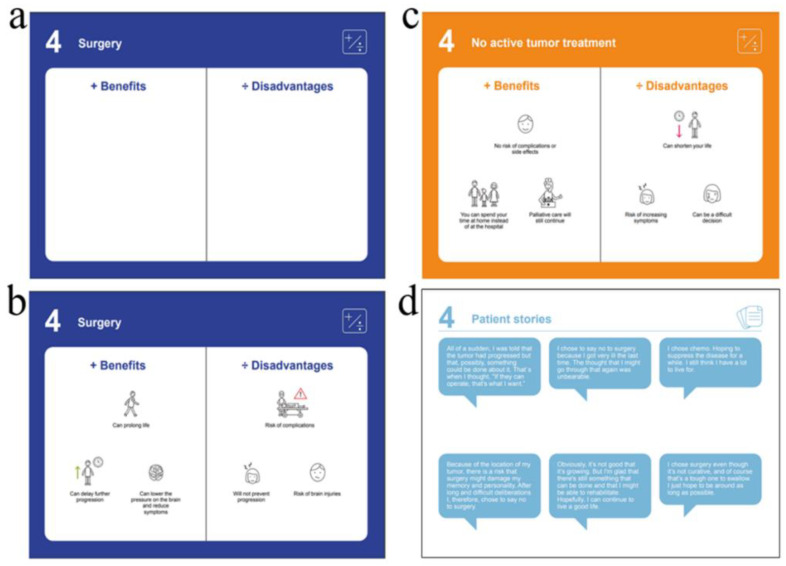
A surgical option card with only the option heading (**a**), the surgical (**b**), and no active tumor treatment (**c**) card populated with the benefits and disadvantages selected during the PtDA development process, and the patient narrative card (**d**).

Step 2. The possible treatment options for patients with recurrent HGG were identified in collaboration with relevant clinicians based on international treatment guidelines elicited from the European Association of Neuro-Oncology (EANO) [3]. We then developed individual option cards for all possible options, including the option of foregoing treatment. The initial cards were equipped with option headings but had no content (Figure 3a). For the patient narrative card, we selected 12 patient narratives from the extensive qualitative data material generated in a previous study on patient and family member decisional needs [22]. 

Step 3. In the first round of the developmental process, the development team was presented with the empty option cards identified in step 2 and asked to suggest the most relevant benefits and disadvantages for each treatment option. The patient and family representatives then evaluated the clinicians’ suggestions and vice versa. All members assessed the 12 patient narratives and the list of preparation sheet questions and selected six patient narratives and six questions. 

Step 4. During the subsequent rounds of processing, the development team members, either individually or in groups, discussed, and refined which preparation sheet questions, patient narratives, and benefits and disadvantages should be presented in the PtDA. This included discussions about wording and pictograms. Before moving forward to the alpha test, we invited a neuropsychologist, a clinical nurse specialist, and two Danish Brain Tumor Association representatives to assess the developed PtDA and preparation sheet. 

### 2.2. Alpha Test

The objective of the alpha test was to assess the acceptability and usability of the PtDA in preparing patients and family members for decision making and supporting clinicians in SDM. 

The alpha test followed three phases: Phase 1: First round of alpha test with clinicians, patients, and family members. Phase 2: Adjustment of the PtDA based on the answers and comments received during the first round of the test. Phase 3: Second round of alpha testing with new clinicians, patients, and family members. 

#### 2.2.1. Participants and Recruitment 

Eligible participants were adult patients with HGG, family members of HGG patients, and clinicians experienced in treating and communicating with HGG patients and their families. Additional inclusion criteria were the ability to speak and understand Danish and the capacity to talk in sentences. Patients with severe aphasia and otherwise eligible participants who had been involved in the development of the PtDA were excluded. Participants were recruited at a large academic hospital through convenience sampling at the oncology and neurosurgical outpatient clinics and through advertising for eligible participants via the Danish Brain Tumor Association’s webpage. At the outpatient clinics, potential participants were screened against the inclusion criteria and informed about the study aim and the subsequent publication of the results by the HSvE. After receiving the information, the participants provided informed consent to participate in the alpha test. The interviews were conducted face-to-face. The participants responding to the online advertisement were informed, and after providing consent, they were interviewed by telephone. All interviews were carried out by the HSvE.

We aimed to include 30 participants, which is equivalent to the “Maximal Process” described in the recent IPDAS update on the development of PtDAs [25]. Additionally, considering the narrow study aim and the structured and theory-guided approach, a sample size of 30 participants, with an equal distribution of patients, family members, and clinicians, was assessed to provide the necessary knowledge and a high degree of information power [28]. Because the involved outpatient clinics did not systematically practice SDM at the time of the alpha test, we defined clinicians as potential users in line with patients and family members.

#### 2.2.2. Data Collection 

Patient and family demographic data were collected concerning sex, age, diagnosis, disease state, and education. For the clinicians, data were collected regarding employment and experience with HGG patients. Data were managed using REDCap electronic data capture tools hosted at OPEN (Open Patient Data Explorative Network, Odense University Hospital, Region of Southern Denmark) [29,30].

To assess the acceptability and usability of the PtDA, we adapted a method proposed by Stacey et al. [31], building on a structured interview guide (Appendix A). The interview guide covered acceptability questions about the amount and balance of the presented information, the understandability of words and pictograms used in the PtDA, and the general perception of and willingness to use the PtDA in clinical practice. The patient and practitioner versions of the Preparation for Decision Making Scale (PDMS) were used to evaluate the usefulness of the PtDA in preparing and supporting participants in decision-making and facilitating consultations [31,32,33]. 

The PDMS was forward-backward translated into Danish in a previous study [34]. It contains 10 items for patients and family members and 11 for practitioners, with 5 response options ranging from 1 (not at all) to 5 (a great deal). One item regarding preparation for follow-up consultations was removed from both the patient and practitioner versions since this item went beyond the scope of the PtDA, leaving nine and ten items, respectively. The items were summed, and the score was converted to a 0–100 scale that presented the perceived level of preparation for decision making [33]. The patient version of the PDMS has previously been validated and has shown very good psychometric properties [32].

The participants were introduced to the PtDA and guided through the decision-making steps, mirroring a natural consultation. Patients and family members could be informed together. After the presentation, the participants responded individually to the structured interview and the PDMS. The interviews were audio-recorded and transcribed verbatim. The PDMS could be answered as either a structured interview or a paper-based questionnaire, depending on the participants’ preferences.

#### 2.2.3. Analysis

The demographic data were analyzed in REDCap using descriptive statistics. The results from the PDMS were transferred to Microsoft Excel (2016), where a two-sided T-test was performed to assess the potential difference between the clinician scores and the patient-family scores. A medical statistician from OPEN-Statistics guided the analysis. 

The transcripts from the structured interviews were transferred to NVivo to ensure rigor and transparency in the analysis. A directed content analysis [27] was carried out with a focus on identifying comments and statements concerning the development of the PtDA and the participants’ perceptions of the PtDA. Codes were developed consecutively. After the second round of alpha testing, the codes from both rounds were merged and divided into themes. Results from the first round of alpha testing were used to adjust the PtDA before the second round.

## 3. Results

### 3.1. Development

In the consultations analyzed to identify preparation sheet questions, 6 of the 10 patients decided on surgery, 4 on chemotherapy without surgery, and 1 on no active tumor treatment. One or more family members were present in 9 out of the 10 cases. During the analysis, seven overall themes were identified (Appendix B): questions concerning details about the treatment options, practical issues, future and prognosis, the surgeon’s opinion, the current situation compared to previous treatments, risk of complications, and features related to the tumor. The development team discussed the themes and decided on six questions to be presented in speech bubbles on the preparation sheet (Figure 2). 

For the in-consult part of the PtDA, six possible treatment options for patients with recurrent HGG were identified: (1) surgery, (2) surgical biopsy, (3) no active tumor treatment, (4) temozolomide, (5) lomustine, and (6) bevacizumab-irinotecan. The majority of the six different option cards presented three benefits and three disadvantages. On the lomustine card, two benefits and two disadvantages were presented, and on the surgical biopsy card, two benefits and three disadvantages were presented. Care was taken to ensure that each option card presented balanced information.

Three option cards were specifically designed for use in the neurosurgical clinic: surgery (Figure 3b), surgical biopsy, chemotherapy in general, and three for the oncology clinic: temozolomide, lomustine, and bevacizumab-irinotecan. The “No active tumor treatment” card (Figure 3c) was designed for use in both neurosurgical and oncological clinics. For the patient narrative card, the development team selected six patient narratives (Figure 3d). These narratives represent two patients deciding on surgery, two deciding to forego surgery, one deciding on chemotherapy, and one patient expressing hope that was unrelated to a specific treatment. The development process proceeded over four rounds, from PtDA version 1 to 5. The fifth version of the PtDA was printed and submitted for the alpha test. 

### 3.2. Alpha Test

#### 3.2.1. Participants

A total of 36 potential participants were approached during the two rounds of the alpha test. Three eligible patients and one family member declined participation due to emotional distress, and one patient was excluded because he did not speak or understand Danish. Thirty-one participants (Table 1) were included in the alpha tests, yielding a response rate of 86%. One patient and one family member were recruited through the Danish Brain Tumor Association. Ten patients, 8 family members, and 11 clinicians were recruited at the neurosurgical and neuro-oncological outpatient clinics.

Among the 11 clinicians, 8 were doctors, and 3 were nurses. Six participants were employed at the Department of Oncology, and five at the Department of Neurosurgery. All clinicians were involved in medical decision making concerning HGG patients every week, and their overall experience with HGG patients ranged between 6 months and 25 years.

The age range of all participants was between 28 and 77 years, and the gender balance was equally distributed. Ten patients were diagnosed with glioblastoma and one with anaplastic astrocytoma. Six patients had experienced recurrence, while five were in their primary treatment or follow-up period. Of the family members, seven participants were partners with the patient, one was a sister, and one was a mother. All participants completed the interview, which lasted 21 min on average (range, 15–47 min).

The first round of alpha testing involved five patients, five family members, and six clinicians; the second round involved six patients, four family members, and five clinicians.

#### 3.2.2. Changes Made to the PtDA during the Alpha Test

Twenty-six participants assessed that the PtDA could be used in consultations as it was. Five participants thought that the PtDA could be used with a few changes. The suggested changes in the first round of the alpha test were as follows: (1) Ensure a visual balance between the benefits and disadvantages by changing some colored details; (2) Create an option card that covered experimental treatment; (3) Remove “treatment in outpatient clinics” from the benefit side of the chemotherapeutic option cards, as transportation time and hospital visits could be considered a disadvantage; (4) Change the pictogram related to “do not prevent progression”.

During the second round, further suggestions were to change the PtDA to an app and let the clinical nurse specialist hand out the patient narrative card to patients and family members instead of having the doctor present it during the decision-making consultation. No changes to the PtDA were made at the end of round two.

#### 3.2.3. Acceptability and Usability

Only minor changes were made to the PtDA between rounds one and two, and the participants’ perceptions of acceptability and usability were comparable in the two rounds. Thus, the results from the two rounds of the alpha tests were summed. All participants found the PtDA to be balanced and neutral, and expressed that it did not favor any option over the other. The text and pictograms used in the PtDA were understandable to 30 participants, while one suggested changing one of the pictograms. Twenty-nine participants believed that the amount of information was appropriate. One participant thought that the PtDA had too little information, and another thought that there was too much. Sixteen out of 20 patients and family members believed that the PtDA could make it easier to make the right decision, and four thought it would make no difference. Ten clinicians were interested in using the PtDA in future decision-making consultations, while one assumed it would be a barrier to communication.

Regarding the PDMS, the converted scores on a 0–100 scale [32] were 65.91 for clinicians (Appendix C) and 80.83 for patients and families (Table 2). These results show that patients and family members assessed the PtDA to have a higher level of perceived preparation for decision making than the clinicians did (*p* = 0.05).

#### 3.2.4. Qualitative Findings

The participants’ qualitative comments concerning the acceptability and usability of the PtDA were divided into four themes.

**1.** A simple design is essential: The visual and tangible format and the simplicity of the PtDA were crucial, especially for cognitively impaired patients. Some participants preferred larger text or more colorful pictograms, while others preferred an all-digital solution.

*“At the third recurrence, your brain is affected pretty bad, so it has to be as simple as possible.”*

(Patient)

**2.** The PtDA might support family involvement: Participants believed that the PtDA, especially the preparation sheet, could encourage discussions between patients and families and prepare them for the consultation. The fact that the in-consult PtDA could be brought home after the consultation was also highlighted as an advantage. One patient, however, emphasized that family involvement was not desirable to all patients and that taking the PtDA home could create difficult conversations.

*“I think it [the PtDA] is a tool for the family as well.”*

(Family member)

**3.** The PtDA must be used correctly: Participants emphasized that the most critical aspects were that the clinician was present and attentive. Several clinicians raised the concern that the PtDA could complicate the consultation, that they did not feel sufficiently equipped to use the PtDA, and that they needed practice. Clinicians who had previously used PtDAs were generally more positive than clinicians with no experience. Most participants believed that if clinicians used the PtDA correctly, it would improve a person-centered approach and strengthen communication between patients, families, and clinicians.

*“I definitely see the advantages. Because it is hands-on. Though I am also a little worried that some clinicians could focus too much on the cards.”*

(Clinician)

**4.** The no-treatment card is important: The no-treatment option card was highlighted to ensure that the option of foregoing treatment was discussed equally with the other options.

*“I think the PtDA might encourage us to make the patients aware that even though we offer them this treatment, they also have the option of saying no to it. And then provide them with the adequate amount of information to decide.”*

(Clinician)

## 4. Discussion

We developed a PtDA following the development steps suggested by IPDAS, and then alpha tested it with potential users. To our knowledge, this is the first PtDA that has been developed to support patients with HGG and their families in decision making at disease recurrence [11,12,17,23]. The participants in the alpha test were generally positive towards the PtDA and its implementation in clinical practice.

Most of the clinicians involved in the alpha test had no experience with SDM or PtDAs, and the SDM steps outlined in the PtDA required clinicians to change their approach to patient communication. Some expressed worries that the PtDA might complicate consultations. The few clinicians with previous experience using PtDAs were more positive toward the PtDA than those without it. These results indicate that a lack of clinical experience and the challenge of having to change the course of consultations might have contributed to clinicians perceiving the PtDA to be less useful than patients and family members perceived it to be. The results also reflect known barriers to SDM implementation [35]. For patients and families, the decision at the time of recurrence directly influences their quality of life and the patient’s survival time. This might explain their readiness for new approaches to decision support and their assessment of the usefulness of the PtDA. Additionally, the simple wording and use of pictograms probably provided more value to patients and families than it did to clinicians. In particular, the cognitively impaired participants in the alpha test underscored the value of a simple design and readily understandable text and pictograms.

The findings are supported by previous research concerning decision support interventions for patients with low health literacy and decision-making challenges due to causes other than brain tumors, which found that pictures increased patients’ understanding of the information provided [36]. A study exploring the effect of presenting glioma patients with a 3D model of their brain and tumor also found that the tangible 3D model positively influenced the patients’ understanding of their situations [37]. These studies underscore the importance of using pictures and a tangible design when developing decision support for patients with cognitive and decision-making challenges.

Participants highlighted the PtDA for its perceived ability to generate and qualify conversations between patients and their families regarding the values and preferences related to the options. This could imply that both patients and family members prefer the family to take on an active and supportive role in decision-making [38]. Thus, considerations concerning the relationship between patient autonomy and family involvement remain important [39]. This aspect is underscored by the fact that one patient in the alpha test preferred not to involve the family in the decision making. To accommodate these different patient perspectives, an individual needs assessment is imperative when providing decision support such as SDM and the use of PtDAs [40]. Another issue to consider when involving family members is the potential discrepancy between the patient’s and the family’s perspectives on prognosis and treatment expectations related to the options [38,39,41,42]. Information preferences may differ between patients and their families [43], highlighting the importance of clinicians eliciting the two parties’ preferences for information and involvement (step 2 in the PtDA). Furthermore, the patient’s dependence on the family may cause an unbalanced power relation [44], which the clinician needs to be aware of during the decision-making process.

One of the option cards in the PtDA presents the option of “No active tumor treatment.” In contrast to the other option cards designed to be used at either the oncology or the neurosurgical outpatient clinic, the “No active tumor treatment” card is designed to be used at both sites. An emphasis on no treatment as a valid option in every treatment discussion is essential in PtDAs and SDM in general [45,46]. Previous research suggests that patients with HGG tend to overestimate the benefits of potential treatments while underestimating their disadvantages, which increases the risk of patients making decisions based on false perceptions [41]. Moreover, advanced cancer patients experience situations where the clinician does not explicitly present the pros and cons of ending active tumor treatment but merely informs these patients about the pros and cons of continuing treatment [46]. This could cause patients to base their decisions on insufficient information and underscore the importance of the clinician delivering honest and realistic information regarding prognostics, benefits, and advantages related to specific options, including the foregoing of active tumor treatment [45,47].

The PtDA in the current study was developed based on a pre-designed Decision Helper template, and this approach offers both strengths and weaknesses. The obvious strength is that the template was developed through a rigorous research process that adhered to the IPDAS criteria and involved a wide range of patient and clinician representatives and designers [26]. The weakness is that the design is fixed, making it harder to adapt the PtDA to a specific clinical situation. The tangible format of the PtDA received positive feedback from potential users, who found it clear and easy to understand. Still, the paper format has limitations regarding distribution and keeping the PtDA up-to-date. Because of this and the increased distribution and use of digital solutions within healthcare, a digital version of the PtDA is currently under development.

The alpha test with potential users was performed face-to-face by the main author and neurosurgical nurse, HSE. This offers the strength that the interviewer is experienced in communicating with HGG patients and family members and a bias in that the included clinicians are colleagues. The clinicians’ answers might have differed had the alpha test been conducted as an anonymous survey or by an unknown researcher. This bias can also be extended to the patients and family members who might have answered the PDMS questionnaire differently in face-to-face encounters than they would have done anonymously. The sample size was limited, and another participant sample might have provided other perspectives. Nevertheless, the 31 participants included in the alpha test equaled the IPDAS’s description of maximal effort, and the variety of the participants strengthened the transferability of the results. By chance, no patients with aphasia were included in the study, and four eligible participants declined participation due to emotional distress. These aspects limit transferability to participants in a relatively good cognitive and emotional state.

Concerning the PDMS, only the 10-item patient version was tested for its psychometric properties. Additionally, we removed one item that was irrelevant to the study objective, and this alteration of the PDMS might have affected its validity. The credibility of the findings was strengthened by data triangulation, with the qualitative data supporting the quantitative results of the PDMS.

The implementation of SDM often requires a change in culture and clinician attitude, and the role of “clinical champions” has been emphasized in previous studies [48,49,50]. Hence, the inclusion of highly recognized clinicians in the development of the current PtDA might offer an advantage in the subsequent implementation process. The alpha test participants underscored that the effect of the PtDA relies less on the design itself and more on the clinicians’ communicative and involvement competencies, which highlights the importance of clinician training as another aspect to include when considering the implementation of the PtDA in clinical practice [48,49,51].

According to the IPDAS recommendations, the alpha test performed in this study should be followed by beta (field) testing before implementing and evaluating the PtDA in larger-scale real-life settings [24,25]. The involvement of cognitively impaired patients in research studies and the decisional needs of HGG patients and their families also require further exploration. With regard to the implementation of SDM with HGG patients, this study shows that emphasis should be placed on keeping decision support simple, tangible, and readily comprehensible to support patients with cognitive impairments engaging in decision making.

## 5. Conclusions

This article describes the development and alpha test of a PtDA developed for use in clinical practice to support SDM and increase decision quality. The PtDA was developed by a multidisciplinary development team and designed for both neurosurgical and oncological consultations. Alpha tests with potential users were carried out in two separate rounds, allowing for changes from the first round to be tested in the second round. Patients, family members, and clinicians were generally positive about the design and use of the PtDA. They expected this to improve decision support and increase SDM for patients with recurrent HGG and their families. It is essential to train clinicians in SDM and on how to use the PtDA. Further evaluation of SDM and the PtDA in real-life decision-making consultations is essential before practice recommendations can be made.

## Figures and Tables

**Table 1:** Demographic characteristics of participants in the alpha test.

Participants	Patients (*n* = 11)	Family (*n* = 9)	Clinicians (*n* = 11)
Male	5	5	5
Female	6	4	6
Age			
20–39	2	1	4
40–59	7	5	7
60–79	2	3	0
Highest education past primary school			
<4 years	4	1	
4–7	5	6	
≥7	2	2	
Patient’s diagnosis			
Glioblastoma	10	8	
Anaplastic astrocytoma	1	1	
The patient’s disease state			
Primary treatment or follow-up	5	4	
Treatment or follow-up after recurrence	6	5	
Clinician demographics			
Department of oncology			6
Department of surgery			5
Doctors			8
Nurses			3
Years of experience with HGG patients			0.5–25

**Table 2:** Preparation for decision making for patients and family members (*n* = 20).

Do You Think the Decision Aid Could:	Not at All 1	A Little 2	Some-What 3	Quite a Bit 4	A Great Deal 5
1. Help you recognize that a decision needs to be made?	0	0	4	2	14
2. Prepare you to make a better decision?	1	2	4	6	7
3. Help you think about the pros and cons of each option?	0	0	1	8	11
4. Help you think about which pros and cons are most important?	0	0	3	4	13
5. Help you know that the decision depends on what matters most to you?	1	0	3	10	6
6. Help you organize your own thoughts about the decision?	1	0	4	7	8
7. Help you think about how involved you want to be in this decision?	0	0	3	11	6
8. Help you identify questions you want to ask your doctor?	1	0	2	3	14
9. Prepare you to talk to your doctor about what matters most to you?	1	0	1	11	7

## Data Availability

Data is contained within the article or in the Appendix A, Appendix B and Appendix C.

## Appendix A. The Structured Interview Guide for the Alpha Test

**Acceptability:**

Balance:

**Do You Think the Presentation of Information in the Decision Helper Is:**	**Yes**	**No**
Clearly slanted towards choosing a specific option?		
Slightly slanted towards choosing a specific option?		
Completely balanced?		
Slightly slanted towards not choosing a specific option?		
Clearly slanted towards not choosing a specific option?		

Do you think the text in the Decision Helper makes sense?
□ Yes □ No
If the answer is no, please elaborate.

Do you think the pictograms in the Decision Helper makes sense?
□Yes □ No
If the answer is no, please elaborate.

Do you think the amount of information in the Decision Helper is:
□ Much less than I wanted
□ A little less than I wanted
□ About right
□ Little more than I wanted
□ Much more than I wanted

Do you think the Decision Helper can be used in consultations?
□ 1, Yes, as it is
□ 2, Yes, but with some alterations
□ 3, No
If the answer is 2 or 3, please elaborate.

Do you think the Decision Helper can make the decision making:
□ Easier
□ Harder
□ Neither easier or harder (no difference)
Please elaborate

Are you willing to use the Decision Helper and/or tell someone about it?
□ Yes □ No
If the answer is no, please elaborate.

Explorative questions:
  What do you like or dislike about the Decision Helper?
Do you have any other comments concerning the Decision Helper?

**Usability:**

Preparation for decision making; Patients and family members

**Do You Think the Decision Aid Could:**	**Not at All** **1**	**A Little** **2**	**Some-What** **3**	**Quite a Bit** **4**	**A Great Deal** **5**
1. Help you recognize that a decision needs to be made?					
2. Prepare you to make a better decision?					
3. Help you think about the pros and cons of each option?					
4. Help you think about which pros and cons are most important?					
5. Help you know that the decision depends on what matters most to you?					
6. Help you organize your own thoughts about the decision?					
7. Help you think about how involved you want to be in this decision?					
8. Help you identify questions you want to ask your doctor?					
9. Prepare you to talk to your doctor about what matters most to you?					

Preparation for decision making; Clinicians

**Do You Think the Decision Aid Could…**	**Not at All** **1**	**A Little** **2**	**Some-What** **3**	**Quite a Bit** **4**	**A Great Deal** **5**
1. Help the patients to fully understand the risks and benefits of the options?					
2. Help the patients identify the importance they place on the risks and benefits of the options?					
3. Help the patients to be as involved in the decision-making process as they desire?					
4. Help the patients to make a more informed decision?					
5. Help you to more fully understand the issues that are most important to the patients?					
6. Help you tailor your counseling to the patient’s preference for decision participation?					
7. Facilitate the consultation?					
8. Affect the patient-physician relationship					
9. Improve the way time is spent during the consultation?					
10. Improve the quality of the consultation?					

## Appendix B

**Table A1:** The process of identifying questions for the preparation sheet.

Examples of Patient and Family Questions Asked during the Consultation	Themes	Representative Questions That Were Selected through Discussions in the Development Team
After the operation, I suppose chemotherapy is an option? Why can’t I get radiotherapy twice?	Details about the treatment options	None
Will I have to do a blood sample the day before I visit the hospital? For how long will [patient name] have to stay at the hospital after the surgery?	Practical issues; time and planning	How much time do I need to spend on transportation and hospital visits?
Last time, he couldn’t be alone when he came home. Will it be the same this time? Can I (the relative) stay at the patient hotel [while the patient is in the hospital]?	Practical issues; family	How will the disease and treatment affect my family?
Will I be ill for two weeks or so? [Chemotherapy] For how long?	Practical issues; daily life	How will the treatment affect my everyday life?
How is it likely to go? It is bad now. What if it gets worse?	Future and prognosis	What will happen if I say no to further treatment?
If you were in my situation, what would you choose?	The surgeon’s opinion	None
I mean, it’s the same [as last time], right? Probably? And the risks are smaller this time?	The current situation compared to previous treatments	How will the treatment course compare to my previous treatment experiences?
What about epilepsy and things like that? Family: Dad, you worry if the surgery will paralyze you, right? Patient: Yeah, we don’t know that, do we?	Risk of complications	What side effects and complications could I experience?
What’s the size of the tumor?	Features related to the tumor	None

## Appendix C

**Table A2:** Preparation for decision making for clinicians.

Do You Think the Decision Aid Could…	Not at All 1	A Little 2	Some-What 3	Quite a Bit 4	A Great Deal 5
1. Help the patients to fully understand the risks and benefits of the options?	0	1	2	8	0
2. Help the patients identify the importance they place on the risks and benefits of the options?	0	1	1	8	1
3. Help the patients to be as involved in the decision-making process as they desire?	0	1	1	8	1
4. Help the patients to make a more informed decision?	0	1	2	5	3
5. Help you to more fully understand the issues that are most important to the patients?	1	0	3	5	2
6. Help you tailor your counseling to the patient’s preference for decision participation?	1	0	2	6	2
7. Facilitate the consultation?	1	0	3	4	3
8. Affect the patient-physician relationship	1	1	4	4	1
9. Improve the way time is spent during the consultation?	1	0	5	4	1
10. Improve the quality of the consultation?	1	0	4	5	1

## References

[1] Louis D.N., Perry A., Reifenberger G., von Deimling A., Figarella-Branger D., Cavenee W.K., Ohgaki H., Wiestler O.D., Kleihues P., Ellison D.W. (2016). The 2016 World Health Organization Classification of Tumors of the Central Nervous System: A summary. Acta Neuropathol..

[2] Stupp R., Hegi M.E., Mason W.P., van den Bent M.J., Taphoorn M.J., Janzer R.C., Ludwin S.K., Allgeier A., Fisher B., Belanger K. (2009). Effects of radiotherapy with concomitant and adjuvant temozolomide versus radiotherapy alone on survival in glioblastoma in a randomised phase III study: 5-year analysis of the EORTC-NCIC trial. Lancet Oncol..

[3] Weller M., van den Bent M., Hopkins K., Tonn J.C., Stupp R., Falini A., Cohen-Jonathan-Moyal E., Frappaz D., Henriksson R., Balana C. (2014). EANO guideline for the diagnosis and treatment of anaplastic gliomas and glioblastoma. Lancet Oncol..

[4] Suchorska B., Weller M., Tabatabai G., Senft C., Hau P., Sabel M.C., Herrlinger U., Ketter R., Schlegel U., Marosi C. (2016). Complete resection of contrast-enhancing tumor volume is associated with improved survival in recurrent glioblastoma-results from the DIRECTOR trial. Neuro-Oncology.

[5] Lu V.M., Goyal A., Graffeo C.S., Perry A., Burns T.C., Parney I.F., Quinones-Hinojosa A., Chaichana K.L. (2019). Survival Benefit of Maximal Resection for Glioblastoma Reoperation in the Temozolomide Era: A Meta-Analysis. World Neurosurg..

[6] Bloch O., Han S.J., Cha S., Sun M.Z., Aghi M.K., McDermott M.W., Berger M.S., Parsa A.T. (2012). Impact of extent of resection for recurrent glioblastoma on overall survival: Clinical article. J. Neurosurg..

[7] Piil K., Jakobsen J., Christensen K.B., Juhler M., Guetterman T.C., Fetters M.D., Jarden M. (2018). Needs and preferences among patients with high-grade glioma and their caregivers—A longitudinal mixed methods study. Eur. J. Cancer Care.

[8] Sterckx W., Coolbrandt A., Clement P., Borgenon S., Decruyenaere M., De Vleeschouwer S., Mees A., Dierckx de Casterlé B. (2015). Living with a high-grade glioma: A qualitative study of patients’ experiences and care needs. Eur. J. Oncol. Nurs..

[9] Elwyn G., Frosch D., Rollnick S. (2009). Dual equipoise shared decision making: Definitions for decision and behaviour support interventions. Implement. Sci..

[10] Musella A., DeVitto R., Anthony M., Elliott Mydland D. (2021). The Importance of Shared Decision-Making for Patients with Glioblastoma. Patient Prefer. Adherence.

[11] Sorensen von Essen H., Piil K., Dahl Steffensen K., Rom Poulsen F. (2020). Shared decision making in high-grade glioma patients-a systematic review. Neurooncol. Pract..

[12] Corell A., Guo A., Vecchio T.G., Ozanne A., Jakola A.S. (2021). Shared decision-making in neurosurgery: A scoping review. Acta Neurochir..

[13] Oliver K., Oliver G., Littlefield B., Knight K., Arons D., Leach D. The Brain Tumour Patients’ Charter of Rights. https://theibta.org/charter/.

[14] Lawler M., Oliver K., Gijssels S., Aapro M., Abolina A., Albreht T., Erdem S., Geissler J., Jassem J., Karjalainen S. (2021). The European Code of Cancer Practice. J. Cancer Policy.

[15] Barry M.J., Edgman-Levitan S. (2012). Shared decision making—Pinnacle of patient-centered care. N. Engl. J. Med..

[16] Elwyn G., Frosch D., Thomson R., Joseph-Williams N., Lloyd A., Kinnersley P., Cording E., Tomson D., Dodd C., Rollnick S. (2012). Shared decision making: A model for clinical practice. J. Gen. Intern. Med..

[17] Stacey D., Légaré F., Lewis K., Barry M.J., Bennett C.L., Eden K.B., Holmes-Rovner M., Llewellyn-Thomas H., Lyddiatt A., Thomson R. (2017). Decision aids for people facing health treatment or screening decisions. Cochrane Database Syst. Rev..

[18] McAlpine K., Lewis K.B., Trevena L.J., Stacey D. (2018). What Is the Effectiveness of Patient Decision Aids for Cancer-Related Decisions? A Systematic Review Subanalysis. JCO Clin. Cancer Inform..

[19] Triebel K.L., Martin R.C., Nabors L.B., Marson D.C. (2009). Medical decision-making capacity in patients with malignant glioma. Neurology.

[20] Martin R.C., Gerstenecker A., Nabors L.B., Marson D.C., Triebel K.L. (2015). Impairment of medical decisional capacity in relation to Karnofsky Performance Status in adults with malignant brain tumor. Neurooncol. Pract..

[21] Hewins W., Zienius K., Rogers J.L., Kerrigan S., Bernstein M., Grant R. (2019). The Effects of Brain Tumours upon Medical Decision-Making Capacity. Curr. Oncol. Rep..

[22] Sorensen von Essen H., Stacey D., Dahl Steffensen K., Guldager R., Rom Poulsen F., Piil K. (2022). Decisional needs of patients with recurrent high-grade glioma and their families. Neuro-Oncol. Pract..

[23] Ottawa H.R.I. Alphabetical List of Decision Aids by Health Topic. https://decisionaid.ohri.ca/AZlist.html.

[24] Coulter A., Stilwell D., Kryworuchko J., Mullen P.D., Ng C.J., van der Weijden T. (2013). A systematic development process for patient decision aids. BMC Med. Inform. Decis. Mak..

[25] Witteman H.O., Maki K.G., Vaisson G., Finderup J., Lewis K.B., Dahl Steffensen K., Beaudoin C., Comeau S., Volk R.J. (2021). Systematic Development of Patient Decision Aids: An Update from the IPDAS Collaboration. Med. Decis. Mak. Int. J. Soc. Med. Decis. Mak..

[26] Olling K., Bechmann T., Madsen P.H., Hugger Jakobsen E., Toftdahl D.B., Hilberg O., Coulter A., Dahl Steffensen K. (2019). Development of a patient decision aid template for use in different clinical settings. Eur. J. Pers. Cent. Healthc..

[27] Hsieh H.F., Shannon S.E. (2005). Three approaches to qualitative content analysis. Qual. Health Res..

[28] Malterud K., Siersma V.D., Guassora A.D. (2016). Sample Size in Qualitative Interview Studies: Guided by Information Power. Qual. Health Res..

[29] Harris P.A., Taylor R., Minor B.L., Elliott V., Fernandez M., O’Neal L., McLeod L., Delacqua G., Delacqua F., Kirby J. (2019). The REDCap consortium: Building an international community of software platform partners. J. Biomed. Inform..

[30] Harris P.A., Taylor R., Thielke R., Payne J., Gonzalez N., Conde J.G. (2009). Research electronic data capture (REDCap)—A metadata-driven methodology and workflow process for providing translational research informatics support. J. Biomed. Inform..

[31] Stacey D., Légaré F., Lyddiatt A., Giguere A.M., Yoganathan M., Saarimaki A., Pardo J.P., Rader T., Tugwell P. (2016). Translating Evidence to Facilitate Shared Decision Making: Development and Usability of a Consult Decision Aid Prototype. Patient.

[32] Bennett C., Graham I.D., Kristjansson E., Kearing S.A., Clay K.F., O’Connor A.M. (2010). Validation of a preparation for decision making scale. Patient Educ. Couns..

[33] Graham I., O’Connor A. User Manual—Preparation for Decision Making Scale. https://decisionaid.ohri.ca/docs/develop/User_Manuals/UM_PrepDM.pdf.

[34] Andersen S.B., Andersen M.O., Carreon L.Y., Coulter A., Steffensen K.D. (2019). Shared decision making when patients consider surgery for lumbar herniated disc: Development and test of a patient decision aid. BMC Med. Inform. Decis. Mak..

[35] Légaré F., Ratté S., Gravel K., Graham I.D. (2008). Barriers and facilitators to implementing shared decision-making in clinical practice: Update of a systematic review of health professionals’ perceptions. Patient Educ. Couns..

[36] Durand M.A., Alam S., Grande S.W., Elwyn G. (2016). ‘Much clearer with pictures’: Using community-based participatory research to design and test a Picture Option Grid for underserved patients with breast cancer. BMJ Open.

[37] van de Belt T.H., Nijmeijer H., Grim D., Engelen L., Vreeken R., van Gelder M., Ter Laan M. (2018). Patient-Specific Actual-Size Three-Dimensional Printed Models for Patient Education in Glioma Treatment: First Experiences. World Neurosurg..

[38] Schumm K., Skea Z., McKee L., N’Dow J. (2010). ‘They’re doing surgery on two people’: A meta-ethnography of the influences on couples’ treatment decision making for prostate cancer. Health Expect..

[39] Ho A. (2008). Relational autonomy or undue pressure? Family’s role in medical decision-making. Scand. J. Caring Sci..

[40] Hoefel L., O’Connor A.M., Lewis K.B., Boland L., Sikora L., Hu J., Stacey D. (2020). 20th Anniversary Update of the Ottawa Decision Support Framework Part 1: A Systematic Review of the Decisional Needs of People Making Health or Social Decisions. Med. Decis. Mak..

[41] Forst D.A., Quain K., Landay S.L., Anand M., Kaslow-Zieve E., Mesa M.M., Jacobs J.M., Dietrich J., Parsons M.W., Horick N. (2020). Perceptions of prognosis and goal of treatment in patients with malignant gliomas and their caregivers. Neurooncol. Pract..

[42] Sharma A., Fruth B., Barrera C., Farfour H.N., Mrugala M.M., Edwin M.K., Sloan J.A., Porter A.B. (2021). How much time do we have? Longitudinal perception of prognosis in newly-diagnosed high grade glioma patients and caregivers compared to clinicians. J. Neurooncol..

[43] Diamond E.L., Prigerson H.G., Correa D.C., Reiner A., Panageas K., Kryza-Lacombe M., Buthorn J., Neil E.C., Miller A.M., Deangelis L.M. (2017). Prognostic awareness, prognostic communication, and cognitive function in patients with malignant glioma. Neuro-Oncology.

[44] Emerson R.M. (1962). Power-Dependence Relations. Am. Sociol. Rev..

[45] Hoffmann T., Beckhaus J., Del Mar C. (2021). ‘What happens if I do nothing?’ A Systematic Review of the Inclusion and Quantitative Description of a ‘No Active Intervention’ Option in Patient Decision Aids. J. Gen. Intern. Med..

[46] Brom L., De Snoo-Trimp J.C., Onwuteaka-Philipsen B.D., Widdershoven G.A., Stiggelbout A.M., Pasman H.R. (2017). Challenges in shared decision making in advanced cancer care: A qualitative longitudinal observational and interview study. Health Expect..

[47] Diamond E.L., Corner G.W., De Rosa A., Breitbart W., Applebaum A.J. (2014). Prognostic awareness and communication of prognostic information in malignant glioma: A systematic review. J. Neuro-Oncol..

[48] Steffensen K.D., Vinter M., Crüger D., Dankl K., Coulter A., Stuart B., Berry L.L. (2018). Lessons in Integrating Shared Decision-Making Into Cancer Care. J. Oncol. Pract..

[49] Joseph-Williams N., Lloyd A., Edwards A., Stobbart L., Tomson D., Macphail S., Dodd C., Brain K., Elwyn G., Thomson R. (2017). Implementing shared decision making in the NHS: Lessons from the MAGIC programme. BMJ (Clin. Res. Ed.).

[50] Joseph-Williams N., Abhyankar P., Boland L., Bravo P., Brenner A.T., Brodney S., Coulter A., Giguère A., Hoffman A., Körner M. (2021). What Works in Implementing Patient Decision Aids in Routine Clinical Settings? A Rapid Realist Review and Update from the International Patient Decision Aid Standards Collaboration. Med. Decis. Mak. Int. J. Soc. Med. Decis. Mak..

[51] Ankolekar A., Dahl Steffensen K., Olling K., Dekker A., Wee L., Roumen C., Hasannejadasl H., Fijten R. (2021). Practitioners’ views on shared decision-making implementation: A qualitative study. PLoS ONE.

